# The bag or the spindle: the cell factory at the time of systems' biology

**DOI:** 10.1186/1475-2859-3-13

**Published:** 2004-11-10

**Authors:** Antoine Danchin

**Affiliations:** 1 Genetics of Bacterial Genomes, Institut Pasteur, 28, rue du Docteur Roux, 75724 Paris Cedex 15, France

## Abstract

Genome programs changed our view of bacteria as cell factories, by making them amenable to systematic rational improvement. As a first step, isolated genes (including those of the metagenome), or small gene clusters are improved and expressed in a variety of hosts. New techniques derived from functional genomics (transcriptome, proteome and metabolome studies) now allow users to shift from this single-gene approach to a more integrated view of the cell, where it is more and more considered as a factory. One can expect in the near future that bacteria will be entirely reprogrammed, and perhaps even created de novo from bits and pieces, to constitute man-made cell factories. This will require exploration of the landscape made of neighbourhoods of all the genes in the cell. Present work is already paving the way for that futuristic view of bacteria in industry.

## Review

### Genomes in the thousands

At the date of October 30^th^, 2004 the GOLD () site provided links to 1205 ongoing or completed genome programmes, most of which from prokaryotic organisms. More than 40,000 pages are indexed in the WWW Browser Engine Google for the keyword "cell factory". Early in 1999 the European Union launched a research programme on the cell factory (), stating that « The concept of the "bio-product" is as old as the knowledge involved in the making of bread, beer, wine or cheese. However, recent techniques and knowledge in molecular biology and genetics mean that living cells – from bacteria to man – are now becoming real "factories". In vast fermentation vats, engineers can direct and control natural metabolism in order to produce all sorts of substances with a high added value: proteins, amino acids, alcohols, citric acid, solvents and even bio-plastics. This industrial mastery of the mechanisms of life opens up revolutionary perspectives in the development of new kinds of medicines, foodstuffs with specific nutritional properties, and biodegradable biochemical products » [1].

Taken together these pieces of information show that exploration of the potential of microbes as industrial tools is shifting from its former status of traditional biotechnology assets to new high technology devices, meant to perform highly specific tasks, with the highest possible yields and security, and genomics as the background support. We shall not here review the use of microbes in traditional production (bread, beer, cheese and wine have been invented since the origin of the Neolithic, and perhaps even earlier [2]) but, rather, see how the coupling between knowledge of bacterial genome sequences and new genomics techniques such as expression profiling and biotechnology processes have interacted recently. The numbers of works in the domain is growing exponentially (it counts certainly in the thousands), and we shall therefore restrict our choice to leads that may be used for further reading. In order to limit the scope of this already extensive study, we shall also restrict this review to cells using the standard genetic code, not considering the extremely interesting attempts to reprogramme the code (for a review see [3]). There is also interesting work developed in vitro, that allows variations on the nature of the building blocks of macromolecules, which will lead to fascinating new aspects of the microbial (artificial) cell factory. We shall not consider this further here (see for example [4-6]).

### General versus particulars

#### Individual genes, from genomics to metagenomics

Genomes are most often viewed as bags of genes. While this is not our personal view – we see genomes as highly organized set of genes, certainly not randomly distributed along the chromosomes [7], this is certainly a limited, but sufficient view for a large area of biotechnology applications. When reading papers emphasizing the biotechnological potential of bacteria, it is not uncommon to witness sentences like that one: « The completion of the *<name of species>*genome sequencing project resulted in the discovery of a number of new genes with potential interest for biotechnological applications ».

Indeed, many biotechnological procedures still rest (and will rest) on the isolation of individual genes, or series of genes involved in the biosynthesis of a specific compound (this is illustrated in the complex case of coenzyme B12 biosynthesis in *Escherichia coli*, for example [8]). Remarkably, it has become a practical fact that it is now much easier (and often significantly less costly) to sequence a genome in its entirety than to isolate a gene of interest and then submit it to mutagenesis for improvement. There are already so many examples of this situation that they cannot be all given here. Many proteases, amylases, lipases and other enzymes of general biotechnological interest (in particular in agro-food industry) are side-products of genes isolated from a variety of genomes [9-11]. As a vivid and lively illustration of the potential of genome programs in the domain of complex molecules such as antibiotics, genes involved in non-ribosomal protein synthesis are continuously collected from genomes, sometimes in an unforeseen way. This was the case, for example with the genome of the entomopathogen *Photorhabus luminescens *[12], that possesses a variety of such highly complex « megasynthases » [13], when it was assumed that most would be present in Gram positives, *Streptomyces *species in particular [13-16], and certainly not in enterobacteria. With this simple gene family expanding exponentially in parallel with the genome programs trend, we need a focused resource to keep track of important developments: the NRPS/PKS database provides us with an updated resource that tries to keep trace of these interesting by-products of genomics certainly promised to a bright future in the domain of chemistry of fine chemicals [17].

The genome concept for identifying new genes of biotechnological interest has now been expanded to that of a « metagenome », formed of communities of organisms (often non-cultivatable) in a given environment [18,19]. This revived the interest for biotechnology of fine chemicals [20], that was proposed for a long time, but remained of limited use. Gene prospecting has already been used to extract interesting variants of genes coding for interesting enzyme activities [21]. There are voices, however, that go against that particular trend, emphasising that the variety provided by artificial means will be much larger than that conceivably produced during evolution [22]. However, although chemistry is extremely efficient, some steps, in particular associated with the chirality of molecules, are costly in terms of the process and its yield. In contrast, chirality is an in-built property of life. We can therefore safely speculate that we shall witness in the near future the use of metagenomics for the revival of biotechnology processes in solving expensive bottlenecks in chemical industrial processes.

However interesting, these « single-gene » approaches remain conceptually very limited, they only explore the surface of what could be provided by the knowledge of genomes. Furthermore, they often aim at the preparation of a single enzyme, that is meant to be used in a process that does not make use of the adaptation and maintenance potential of living organisms. In short, the cell is not used for what it is in reality, a factory. This is however dramatically changed with the advent of genomics as we shall now see.

### Functional genomics

Progresses in genome sequencing were followed by attempts to better understand how a cell behaves as a whole. The knowledge of complete genome sequences permitted scientists to set up expression profiling techniques that play an ever increasing role in biotechnology [23]. Indeed the corresponding knowledge can be used, when the genome of a bacterium used in industry is known, to improve its behaviour, stability, yield in production or security [24].

Many metabolic engineering strategies now use genome-wide methodologies such as DNA sequencing, transcription profiling and global analysis of metabolites. These techniques allow the identification of genetic differences and provide insight into their cellular effects. Inverse metabolic engineering endeavours to map differences between strains with different degree of a certain desired phenotype and subsequent identification of factors conferring that phenotype. Briefly reviewed, expression profiling can be divided into three major branches that each have a particular outcome, and gives a specific knowledge on the organism.

#### The transcriptome

With all genes known from a cell it has been possible to create DNA arrays sampling a subfamily or all genes on a variety of physical supports. These arrays can subsequently been used to monitor the level of expression of each gene in a particular condition. While this transcriptome approach is widely used, its interpretation is still a matter of research [25,26] but is continuously improving [26,27]. Indeed the very fact that an experiment has, embedded in the data, a collective behaviour is until now rarely used as such, while multifactorial analysis techniques would certainly provide new insights [28]. However transcriptome expression profiling has already had considerable impact in biotechnology. A case in point is improvement of lysine production, despite the fact that this amino acid has previously been manufactured using bacteria for more than 40 years [29].

#### The proteome

The second level of expression profiling is, of course, that of the direct access to the gene products, the proteins. Two-dimensional gel electrophoresis has been developed for thirty years, with considerable success, but it is still extremely limited by the lack of reproducibility of 2-D gel patterns [30]. Other methods try to by-pass the 2-D gel step by direct coupling of high-performance mass spectrometry instrumentation with highly efficient chromatographic and electrophoretic separations [31]. While it is a method of choice for qualitative studies, the latter however are usually difficult to use when one wishes to compare the outcome of several experiments. 2D-gel electrophoresis appears therefore to have still a bright future in the domain. Proteomic studies are complementary to transcriptome analysis [10,32-35], because translation efficiency is variable [36,37], and because mRNA stability can also vary [38,39]. They are just beginning to demonstrate their importance in the study of complexes that organize the cell factory [40].

#### The metabolome

Fermentation processes often aim at producing a given metabolite. The major problem facing industry in this domain is to improve the production yield, often for products that do not have a very high added-value (as opposed to proteins used in medicine, for example), in a background that has already been improved by generations of mutational improvement. Furthermore, many metabolites have to be as pure as possible, trying to prevent contamination by side-products that may be toxic [41]. It is therefore of importance to be able to analyse the whole metabolite set of cultures growing in a variety of conditions, and to relate it to gene expression [42,43], so that educated guesses may be explored for improvement of the processes of interest [44]. While there is currently no efficient large-scale way to systematically monitor metabolites in cells (Nuclear Magnetic Resonance, for example, is limited by its poor sensitivity to those metabolites that are at a high concentration in the cell and Mass Spectrometry needs preliminary purification steps to sort out the zoo of molecules generated in a cell) "metabolomics" is one of the most fashionable "omics" at present [45,46]. It has already been used efficiently in the case of focused production, such as synthesis of antibiotics [47]. There is little doubt that this domain will expand considerably in the near future [45].

### Gene expression and genome organisation

The traditional way for biotechnology to improve its processes was to select mutants having interesting properties (in terms of stability, resistance to foreign agents such as viruses, and of course metabolite or biomass production). This required long and tedious procedures where relevant features were usually gradually improving [48]. However these slow changes had a remarkable, although unobtrusive, consequence. Rather than involving isolated mutations, in many cases a coordinate set of mutations was improving the quality of production. Unfortunately, in the absence of direct access to the genome sequence, it was not possible either to identify or to tell those which were important and those which were dispensable. Furthermore, even when the sequence is known it is far from being straightforward to tell, from the differences observed with the parent strains, what are the important ones. Genomics, with all its "omics" complements, nevertheless completely changed the picture, and it is now possible to optimise production knowingly, using molecular targets that are directly extracted from knowledge of the genome. This has been applied for example in the case of the much studied *Corynebacterium glutamicum *[49].

Further progress is certainly possible. It is important to try to understand whether genomes are simply random collection of genes, or whether they show rules, that might be exploited for using cells as factories. Remarkably, at least in bacteria, the organisation of the genome reflects some kind of optimisation of gene expression [50]. Genes do not work in isolation, and their products, even in bacteria, are likely to be compartmentalised. The study of the landscape of all neighborhoods of a gene (proximity in the chromosome, codon usage bias, phylogeny of its products, electric charge, amino acid composition, participation in complexes, and even a neighborhood benefiting from the expertise of other scientists, such as the co-occurrence of gene names in a same article – "in biblio") provides a systemic view that must be used to optimise the behavior of the cell [51].

While this has not yet, to our knowledge, be taken into consideration for improvement of production by industrial strains, it is more than likely that this will be performed in the near future (in fact it is likely that optimisation of the global properties of gene or gene islands text has already been used for the industrial production of proteins, but because protection by patenting is difficult, if this has been done the corresponding know-how is likely to be protected by secrecy). Regulation of gene expression is also of major interest. It must be understood however that this feature of life is evolving much more rapidly than catalytic or structural components of the cell. One should therefore be cautious in extrapolating knowledge from an organism to another one. Theoretical studies, associated to validation experiments have now begun to decipher the rules that govern regulation of gene expression, and it is certainly already possible to construct subtle regulation systems [52], that are much more sophisticated than the ubiquitous on/off systems using positive or negative control of transcription [53-56].

Among the recent discoveries that will play a considerable role in genome-mediated control of gene expression is that of riboswitches [57]. This mode of control seems to be ubiquitous, but significantly different between Gram negatives and Gram positives (where it appears to be more widely spread). It is still early to have an exact idea of the impact on industrial processes, but the very fact that many coenzymes (vitamins) biosynthetic pathways are controlled using riboswitches warrants further exploration.

In the same way quorum-sensing has much to say for the control of gene expression at high cell density [58]. Until now this general control process – which is still under investigation – has not been explicitly used to control production in cell factories. It seems likely that, once deciphered in its details, it will be a parameter introduced in large-scale productions. The recent serendipitous discovery that borate was involved in the construction of the mediator AI-2 (autoinducer-2) demonstrates however that unexpected features should always be considered as a possibility when a process does not go entirely as planned [59].

### Model cell factories

Many bacteria have been used as cell factories. In most cases this was to produce small molecules (in particular antibiotics, vitamins and amino acids). These bacteria were usually the result of continuous improvement using standard mutagenesis/screening techniques of bacteria isolated in the wild. Streptomyces species, for example, account for a large number of antibiotics production. *Streptomyces lividans *[60], *Corynebacterium glutamicum *[61], *Bacillus subtilis *[24], *Escherichia coli *[62], *Zymomonas mobilis *[63], to give a few names, are used not only as models but also as large-scale production factories. Perhaps the largest scale production fermenters (often 150 m^3^) are now growing *Xanthomonas campestris*, used as a supplement not only in food, but also in dentrifrice, housekeeping products and even in painting, to prevent it from making drops [64]. In the past decades even larger fermenters were used to produce biomass or ethanol, a trend that was abandoned with cheap oil prices, but that will most probably resume its older importance as the price of oil rises sharply.

Among those, most bacteria were chosen for their industrial purpose, as a prime intention. As a consequence, until the advent of genome programs, they were only known for their physiological and physico-chemical properties in fermenters, with limited knowledge of their genetic properties. This was such an inconvenience that, very early on, industry explored the usability of the universal model *E. coli *as a ubiquitous cell factory. This trend was particularly emphasized as soon as genetic engineering techniques were developed, as early as in the early eighties, with the construction of new vectors for expressing foreign proteins at will (e.g. [65]). Mid-eighties many proteins of medical interest were produced using *E. coli *as the factory. This is still so today, with only little shift to the use of other bacterial species as factories. This was initially limited to high added-value products, allowing for very expensive purification steps and compliance to very tight regulations. In quite a few cases however *E. coli *and sometimes other model bacteria, in which appropriate genes were introduced either on plasmids or in the chromosome, was highly efficient in producing low cost metabolites [66]. *Escherichia coli *is even used in the production of amino acids, in industrial quantities, a production that was initially reserved to specific mutants of species that had been slowly improved over the years.

GRAS organisms, such as AT-rich Gram positives, such as *B. subtilis*, are much more difficult to use, except for biomass production, or secreted proteins, because heterologous protein expression is difficult there. This has been understood after the genome was deciphered, as a consequence of the poorly versatile control of translation initiation (lack of ribosomal S1 protein in particular [67]), as well as of the large number of proteases harbored by the organism [68].

Taken together these observations suggest the rational choice of a new organism that would play the role of a ubiquitous cell factory. This organism should have several properties. It should be non pathogenic, and its envelope should not trigger inflammation reactions in animals (Man included). It should be easily transformable and allow recombination with linear DNA, with as little matches needed for recombination as possible. It should grow fast at temperature compatible with the size of fermenters (metabolic activity heats up the medium), and it should reach high cell density. More specialized views, adapted to specific productions will also be considered at some point, but it will be interesting to witness the choice of new model bacteria in the new era of the cell-factory.

## Conclusions

Bacteria have been used as factories for a long time. A first step to rationalize this approach has been met with the first genetic engineering of *E. coli*, producing heterologous proteins. As we now sequence a new genome every third day or so, it is clear that we will be able soon to understand the core of bacterial life, and probably be able to choose new models, better suited to the goals of industry. However we must always remember that life is full of surprises, even in the best explored domains: who would have thought that *E. coli *communicates with its kins using the boron atom? Discovery cannot be planned, and the most surprising observations, that have the most considerable consequences in terms of applications of research, come from studies that are totally academic in nature (who would have thought that the discovery of RNAi would have come from the study of variagation in petunia flowers?). One should not mix up domains: discovery first, and this needs a considerable degree of freedom of choice in the topics explored, and then, naturally, one can think of applications of research. Constructing the best of bacterial cell factory would be such a goal.

## List of abbreviations

GRAS: Generally Recognized As Safe

RNAi: RNA interference

## Authors' conflict of interest

The author declares that he has no competing interests.
